# Alantolactone triggers oxeiptosis in human ovarian cancer cells via Nrf2 signaling pathway

**DOI:** 10.1016/j.bbrep.2023.101537

**Published:** 2023-09-07

**Authors:** Mahdieh Nasirzadeh, Somayeh Atari Hajipirloo, Shiva Gholizadeh-Ghaleh Aziz, Yousef Rasmi, Ghader Babaei, Shahriar Alipour

**Affiliations:** a Cellular and Molecular Research Center, Cellular and Molecular Medicine Institute, Urmia University of Medical Sciences, Urmia, Iran; b Department of Clinical Biochemistry, Faculty of Medicine, Urmia University Medical Sciences (UMSU), Urmia, Iran; c Department of Clinical Biochemistry and Applied Cell Sciences, Faculty of Medicine, Urmia University of Medical Sciences, Urmia, Iran; d Student Research Committee, Urmia University of Medical Sciences, Urmia, Iran

**Keywords:** Ovarian cancer, Alantolactone, Oxeiptosis, Nrf2 signaling pathway

## Abstract

**Introduction:**

A growing body of evidence indicated that Alantolactone (ALT) promotes Reactive Oxygen Species (ROS) generation exclusively in cancer cells. Therefore, the aim of this study was to investigate the effect of ALT on the molecular mechanism of oxeiptosis, as a novel cell death pathway due to the high levels of intracellular ROS in ovarian cancer.

**Methods:**

MTT assay was used to evaluate the effect of ALT on SKOV3 cell viability. mRNA and protein expression levels of Nrf2 (nuclear factor erythroid 2–related factor 2), KEAP1 (Kelch-like ECH-associated protein 1), PGAM5 (phosphoglycerate mutase family member 5), AIFM1 (Mitochondrial Apoptosis-Inducing Factor), Glutathione synthetase (GSS) and glutathione peroxidase (GPX) were analyzed by real time PCR and western blotting methods respectively.

**Results:**

Our findings showed that ALT inhibits the proliferation of skov3 cells in a time and dose dependent manner and IC50 was 32 μM at 24h.

A significant down-regulation of Nrf2, GSH and GPX mRNA levels was seen in skov3 cells incubated with 32 and 64 μM of ALT in comparison with control group, while, mRNA expression levels of PGAM5 and KEAP1 were increased.

Western blot analysis showed that ALT significantly decreases protein levels of Nrf2 and increases PGAM5 and KEAP1.

ALT dephosphorylated PS116-AIFM1 and total AIFM1 protein level was elevated.

**Conclusion:**

Our results provided evidence that ALT could be a potential option for ovarian cancer treatment by ROS-mediated oxeiptosis.

## Highlights

• ALT inhibits ovarian cancer cell proliferation.
• ALT induce ROS overproduction by suppressing Nrf2.
• ROS elevation was followed by PGAM5 upregulation.
• PGAM5 dephosphorylate AIFM1 and induce oxeiptosis cell death pathway.

## 1 Introduction

Ovarian cancer is one of the most lethal gynecological malignancies in the female reproductive system. Statistically, this cancer is the eighth most common cancer in women, and 300,000 serious cases are diagnosed annually, which is responsible for 3.4% of all female cancers [1,2]. Mounting evidence indicated that ovarian cancer is often diagnosed in advanced stages due to its high metastatic and asymptomatic nature and therefore has a high mortality rate [[3], [4], [5]]. Therefore, the combination of optimal tumor surgery and chemotherapy is an applicable treatment for ovarian cancer [6]. The therapeutic strategy currently used to treat patients with ovarian cancer is often associated with compensatory side effects. For this reason, in recent years, significant research has been conducted to identify new drugs with therapeutic potential in the treatment of this cancer. Among these, natural compounds have attracted the attention of many researchers due to their broad structural and functional features as well as fewer side effects [7].

Alantolactone (ALT) is one of the most important members of sesquiterpene lactone, which has wide variety of biological properties such as anti-bacterial, anti-inflammatory and anti-cancer features [8,9]. In recent years, research has suggested that ALT exhibits anti-tumor potential against various types of cancers, including human colorectal cancer [10], liver cancer [11,12], leukemia [13], breast cancer [14], lung cancer [15,16] and cervical cancer [17]. Hyper activation of Nrf2 has been identified in diverse cancer tissues [[18], [19], [20], [21], [22], [23], [24]], including lung [25,26], pancreas [27] and endometrium [28]. Some flavonoids similar to chrysin, apigenin, luteolin and 4-Methoxychalcone decrease Nrf2 molecular action by stimulating the degradation of mRNA and protein levels of Nrf2 and inhibit cancer cells to survive chemotherapy [[29], [30], [31]]. Epigalocatechin3-gallate down regulates Nrf2 –HO-1 axis as an anticancer effect [32]. Intriguingly, various human cancers usually express boosted levels of Nrf2. Nrf2 inhibitors make the cancer cells more sensitive to anticancer chemotherapeutic drugs via inhibition the activity of drug excretion transporters and detoxification enzymes [24,33].

One of the mechanisms by which ALT exerts its anti-cancer activity is the reduction of glutathione (GSH) or inhibition of thioredoxin reductase (TrxR), which leads to the accumulation of ROS in cancer cells [8]. For example, Junmin Zhang and colleagues showed that ALT cause cell death in cervical cancer cells by inhibiting thioredoxin reductase and accumulation of intracellular ROS levels [34]. In another study, Muhammad Khan et al. reported that ALT depletes GSH by direct conjugation to GSH, eventually leading to intracellular ROS accumulation and cell death in hepatocellular carcinoma [35].

Extensive studies on the cell response to ROS show that KEAP1, as the main ROS sensor in the cell, generates different responses according to the quantity of intracellular ROS.

At low ROS levels, KEAP1 conformation changes, leading to release of the nuclear factor erythroid 2–related factor 2 (Nrf2) and its translocation into the nucleus, and ultimately increasing the expression of cytoprotective genes to protect the cells from oxidative stress. On the other hand, at high levels of ROS, the interaction between KEAP1 and PGAM5 is interrupted and a phenomenon called Oxeiptosis occurs. Oxeiptosis is a novel type of radical-induced caspase-independent cell death pathway that occurs through activation of the KEAP1-PGAM5-AIFM1 signaling pathway at high ROS levels [36].

However, the ATL-induced ROS accumulation and related molecular signaling pathways in cancer cell death have not been clearly revealed.

Understanding ALT induced ROS overproduction and its correlation with oxeiptosis pathway can be helpful in finding new approach in cancer therapy strategies based on natural compounds such as ATL.

## 2 Materials and methods

3-(4,5-dimethyl-2-thiazolyl)-2,5-diphenyl-tetrazolium bromide (MTT) and dimethyl sulfoxid (DMSO) were procured from Sigma (USA) and Merck (Germany), respectively. Alantolactone (ATL: cat. No: SML0415-Germany) was initially dissolved in DMSO to prepare stock solution and was stored in -20 °C until it was used. Fetal bovine serum (FBS), penicillin-streptomycin and Trypsin-EDTA were provided from Biowest (France). Primary antibodies against β-Actin (sc-47778), Nrf2 (sc-365949), KEAP1 (sc-365626), PGAM5 (sc-515881) were purchased from Santa Cruz Biotechnology (USA). The antibody against AIFM1 (E-AB-10123) was purchased from Elabscience biotechnology.

The antibody againstAIFM1 (Ser-116), phospho-specific (Cat. # AP5501) was purchased from ECM bioscience. All experiments were performed in triplicate.

### 2.1 Cell culture

The human ovarian cancer cell line (SKOV3) was purchased from National Cell Bank of Iran (NCBI) (Tehran, Iran), and was cultured in DMEM-high glucose medium supplemented with 10% (v/v) heat-inactivated FBS, 100 units/mL penicillin, and 100 μg/mL streptomycin, and incubated at 37 °C in a humidified incubator (95%) in a 5% CO2 atmosphere. Subculture was performed 3:1 when the cells reached 80–90% confluence in T25 cell culture flasks. The experiments were done in subculture 3-6th of SKOV3 cell lines.

### 2.2 MTT assay

MTT Assay was used to evaluate cell viability. The cells were cultured at a density of 5 × 10^3^ cells per well in 96-well plates. After 24h, the cells were treated with various concentrations of ATL (1, 5, 10, 25, 50, 75, 100 and 150 μM) for 24, 48 and 72 h. SKOV-3 cells treated only with cell medium were used as the control. 20 μL of 5 mg/mL MTT solution was added to each well and then the plates were kept at 37 °C for 4 h. After eliminating MTT solution, cells were incubated with DMSO (150 μL) on a plate shaker at 37 °C for 10 min and the optical density (OD) was measured at 570 nm (630 nm as a reference) by a micro plate reader (BioTek, USA) and relative cell viability was stated as percentage of non-treated controls as follows:

Cell viability (%) = (A570sample - A570blank) / (A570control - A570blank) x 100

Half maximal inhibitory concentration (Ic50) was determined by Compusyn software (Combosyn, Inc., Paramus, USA).

### 2.3 Protein extraction and western blotting analysis

The SKOV3 cells were incubated with ALT (0, 16, 32 and 64 μM) for 24 h. Then they were gathered and washed twice with PBS for preparing to lyse in lysis buffer (50 mM Tris–HCl at pH 7.5, 150 mM NaCl, 0.5% sodium orthovanadate (Na3VO4 0.5% sodium deoxycholate, 50 mM EDTA, 50 mM NaF, 1% Triton X-100, 0.1% SDS) mixed with protease inhibitors (1 mM phenylmethylsulfonyl fluoride, 0.5 mM aprotinin, 0.5 mM leupeptin). The protein concentration in the supernatant was measured based on Bradford method [37]. Proteins were separated by 12% SDS-PAGE after electrophoresis and then were electrotransferred to polyvinylidene fluoride (PVDF, Millipore) membranes, and were blocked by 3% BSA in TBST buffer (20 mM Tris–HCl pH 7.5, 150 mM NaCl and 0.1% Tween-20) for 2 h at room temperature. The membranes were then probed with diluted primary antibodies (Nrf2, KEAP-1, PGAM5, AIFM1 and AIFM1 (Ser-116), phospho-specific) in 1% BSA/TBST (overnight, 4 °C), and were washed triplicate with TBS-T buffer for 10 min, then were incubated with secondary antibodies conjugated by horseradish peroxidase (HRP) (1:2000,Cat no: 1105A211) for 2 h at room temperature and were washed extensively three times with TBS-T buffer for 10 min before being detected by chemiluminescence with the ECL kit (Cat no: CMGECL). The membrane was rinsed in water to remove excess chemiluminescent substrate on the membrane. Then membrane protein-side up was incubated in the harsh stripping buffer (6 M GnHCl, 0.2% NP-40, 100 mM β-mercaptoethanol, 20 mM Tris–HCl, pH 7.5) with gentle agitation, for 30 min at room temperature then washed thoroughly 4 times with PBS containing 0.1% Tween-20. After stripping, membrane was tested again with different antibodies where needed.

Ultimately, proteins were revealed by exposing the blots to film and quantified western blotting data by Image J Software densitometry.

### 2.4 RNA extraction and cDNA synthesis

Total RNA extraction from investigated cells were performed with GeneAll HybridRTMRNA extraction kit (Seoul, South Korea). The purity and concentration of extracted RNA were evaluated by NanoDrop TM 2000 Spectrophotometer (Thermo Fisher Scientific, USA). The cDNA was synthesized by using SuperScript III™ First Strand synthesis kit (GeneAll, South Korea).

### 2.5 Real-time PCR

Primer sequences were designed according to the melting temperature and were checked for primer dimer and hairpin formation with Oligo7 primer analysis software (Table 1). Quantitative real-time PCR was done using Real Q Plus 2x Master Mix Green (Amplicon, Denmark) and Bio Molecular Systems MicqPCR cycler (QLD, Australia). The PCR thermal schedule were as follows: 15 min at 95 °C, then 40 cycles of 95 °C for 20 s; 58 °C for 60 s and 72 °C for 5 min. The relative expression levels of Nrf2, KEAP1, PGAM-5, GSH and GPX genes were normalized using β-actin as a reference gene. The 2^-ΔΔCt^ method was used for comparative quantification.

**Table 1:** Nrf2, GSH, GPX, KEAP-1, PGAM5 and β-actin primer sequences that were used to evaluate gene expression in Real-time PCR.

Primer name	Primer sequence
Nrf2 Forward	5′ TTCACTAAACACAAGTCCCAGT 3′
Nrf2 Reverse	5′ CAGGGGCACTATCTAGCTCT 3′
GSH Forward	5′ TCATTCCTCTCCTAGCCCTCC 3′
GSH Reverse	5′ AGACACAACTTTTCTGGTCCT 3′
GPX Forward	5′ CCCTCCTACCCCGGCTGCTGCTTG 3′
GPX Reverse	5′ ACAGGACCAGCACCCATCTCG 3′
KEAP-1 Forward	5′ GTGTCCATTGAGGGTATCCAC 3′
KEAP-1 Reverse	5′ TGGTACATGACAGCACCGTTC 3′
PGAM5 Forward	5′ CTTCATCTGGGTTTTTGCTTT 3′
PGAM5 Reverse	5′ CTCGGCCCCTAAAGACCT 3′
β-Actin Forward	5′ CTGGAACGGTGAAGGTGAACA 3′
β-Actin Reverse	5′ TGGGGTGGCTTTTAGGATGG 3′

## 3 Statistical analysis

Data analyses were performed with GraphPad Prism Software version 9.0(GraphPad Software Inc., La Jolla, CA) using one-way ANOVA with Tukey's test. The p<0.05 was considered statistically significant.

## 4 Results

### 4.1 ALT inhibited the proliferation of SKOV-3 ovarian cancer cells

**Fig. 1:**
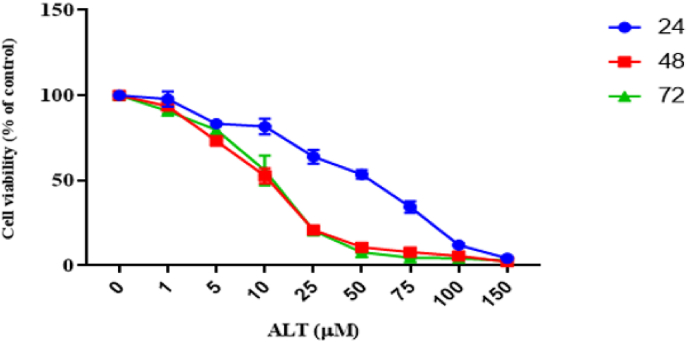
ALT promotes cell death of SKOV-3. SKOV-3 cells were treated with various concentrations of ALT during 3 different time (24, 48 and 72 h). Cell viability was determined by the MTT assay.

MTT assay was done to survey the anti-proliferative effects of ALT on ovarian cancer cells proliferation and survival. SKOV3 cells were incubated with various concentrations of ALT (0, 1, 5, 10, 25, 50, 75, 100, and 150 μM) for 24, 48, and 72 h. Our findings showed that, in comparison with the control group cells, ALT greatly suppresses the proliferation of SKOV3 cells in a time- and dose-dependent manner. (Fig. 1). Half maximal inhibitory concentration (Ic50) was 32 μM at 24 h, 9.66 μM at 48 h and 8.05 μM at 72h. Based on these results 16, 32 and 64 μM of ALT were used for the subsequent experiments.

**Fig. 2:**
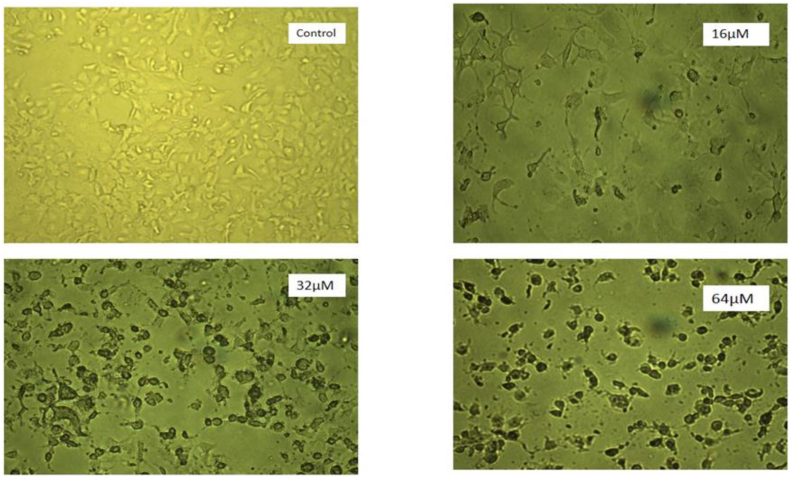
Morphological changes of SKOV3 cells treated with different concentrations of ALT: cells were treated with ALT (16, 32 and 64 μM). After 24 h incubating, modulation of cells form was observed by an inverted light microscope.

Morphological data also confirmed that ALT treatment significantly inhibits the cell proliferation of SKOV3 cells (Fig. 2).

### 4.2 Effects of ALT on Nrf2, GSH, GPX, KEAP1 and PGAM5 mRNA expression in SKOV-3 cell line

**Fig. 3:**
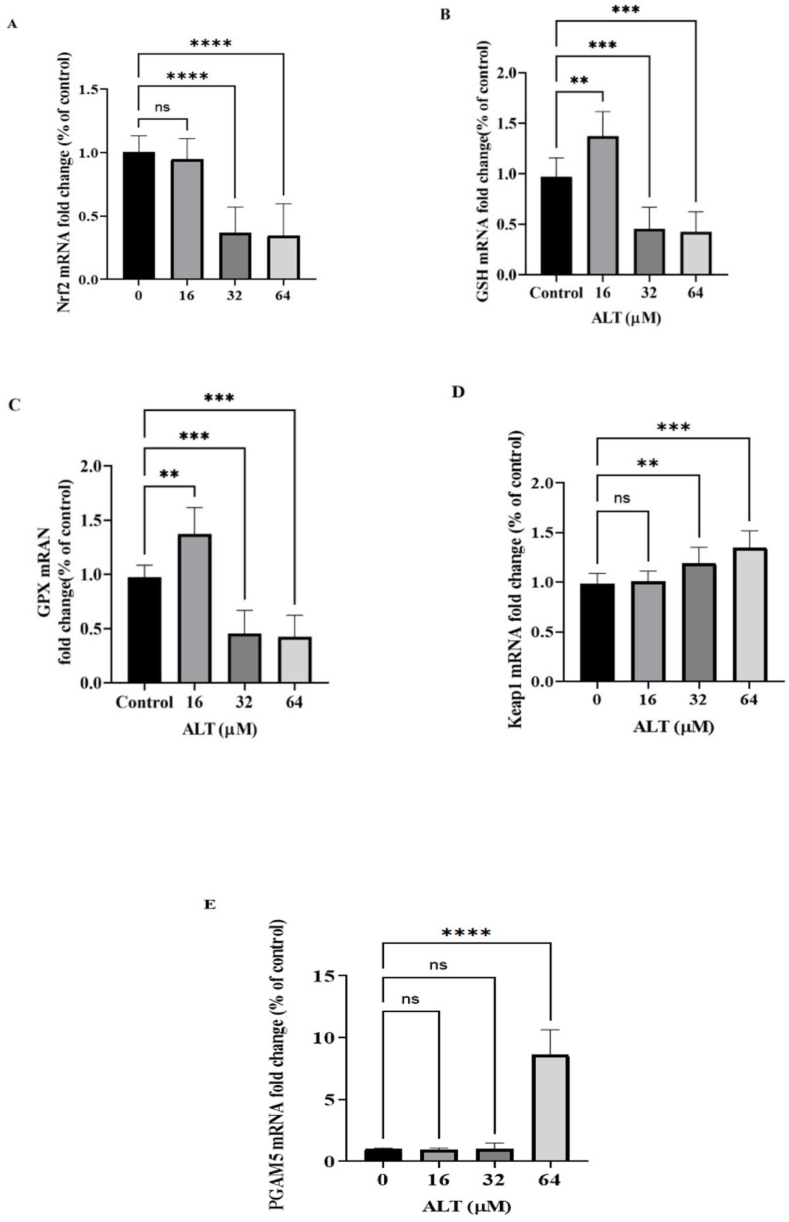
Real-time RT-PCR analysis reveals effect of ALT on mRNA expression of **A.** Nrf2, **B.** GSH, **C.** GPX, **D.** KEAP1 and **E.** PGAM5; SKOV-3 cells were incubated with IC50, 0.5 × IC50 and 2 × IC50 concentrations of Ala for 24 h. Expression of mRNA levels are presented (normalized to β-actin). Data explicited as mean ± SD of three independent experiments. The significance was assessed with a one-way ANOVA test. For p-value each symbol is shown significance to control group. (*p < 0.05, **p < 0.01, ***P < 0.001, ****P < 0.0001).

As presented in Fig. 3-A, significant down regulation of the Nrf2 mRNA levels were observed in SKOV-3 cells incubated with 32 and 64 μM of ALT in comparison with control group.

ALT decreased significantly the mRNA expression levels of GSH (Fig. 3-B) at 32 and 64 μM, whereas there was a significant increase in 16 μM group. This appears to be consistent with this fact that mild-levels of ROS could activate the transcription of antioxidant enzymes such as GSH.

The mRNA expression levels of GPX decreased significantly in a dose dependent manner (Fig. 3-C). These results showed the toxic effects of ALT on ovarian cancer cells through the reduction of antioxidant power which leads to an increase in intracellular ROS.

Treating cells with ALT results in increased KEAP1 expression, and the rise is significant at the 64 μM concentration. These notable results showed that the up regulation of KEAP1 occurs in line with the down regulation of Nrf2. (Fig. 3-D).

ALT increased significantly mRNA expression levels of PGAM5 in 64 μM treatment group in comparison to control group (Fig. 3-E). Upregulation of KEAP1 and PGAM5 is associated with down regulation of Nrf2. This suggests a relationship between three components and so we used western blot technique to confirm this theory.

### 4.3 Effects of ALT on Nrf2, KEAP1, PGAM5, AIFM and AIFM1 (Ser-116), phospho-specific protein levels

**Fig. 4:**
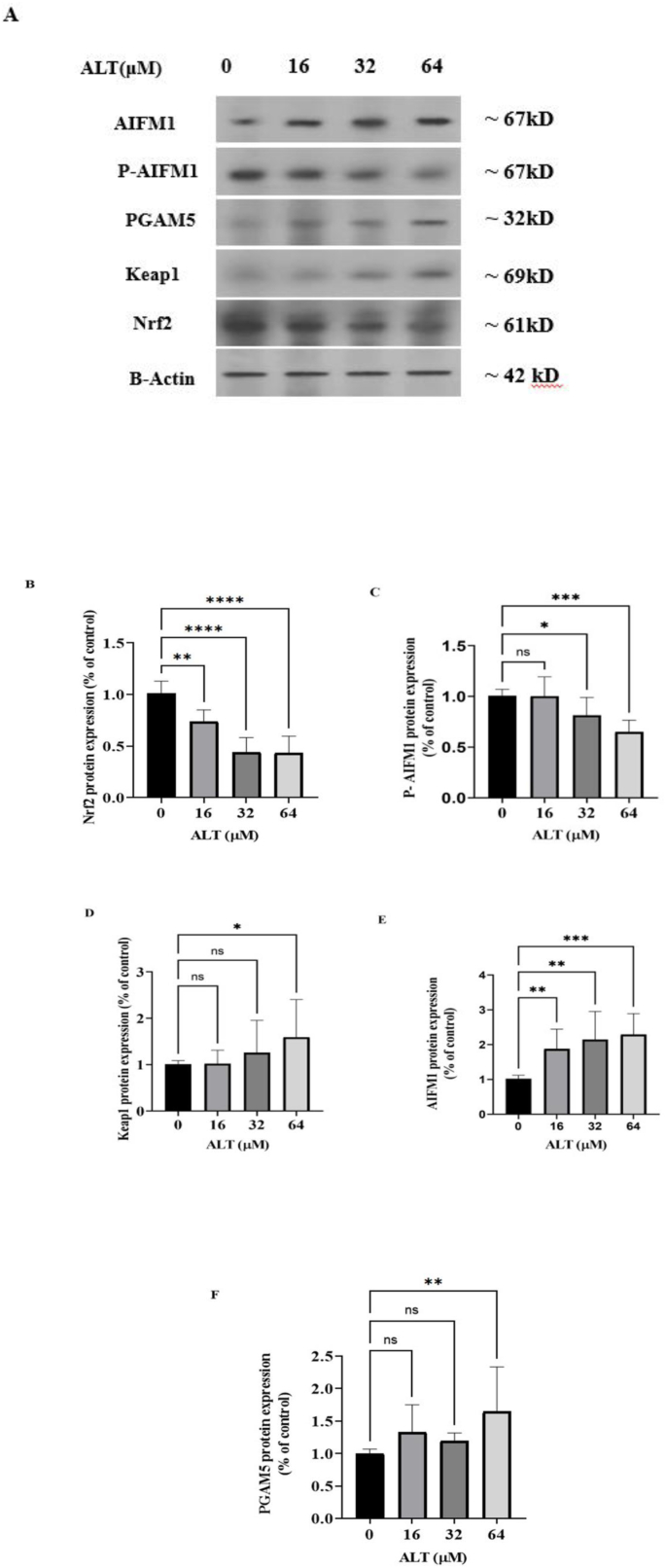
Western blotting shows KEAP1-PGAM5-AIFM1 pathway protein level regulation by ALT. SKOV-3 cells were treated with different concentrations (0, 16, 32 and 64 μM) of ALT for 24 h. Western blot images (**A**) analysis showed that Ala decreased protein levels of **B.** Nrf2 and **C.** P-AIFM1(Ser-116), phospho-specific. ALT increased protein levels of **D.** KEAP1, **E.** AIFM1and **F.** PGAM5. Data expressed as mean ± SD of three independent experiments. The significance was assessed with a one-way ANOVA test. For p-value each symbol is shown significance to control group. (*p < 0.05, **p < 0.01, ***P < 0.001, ****P < 0.0001).

**Fig. 5:**
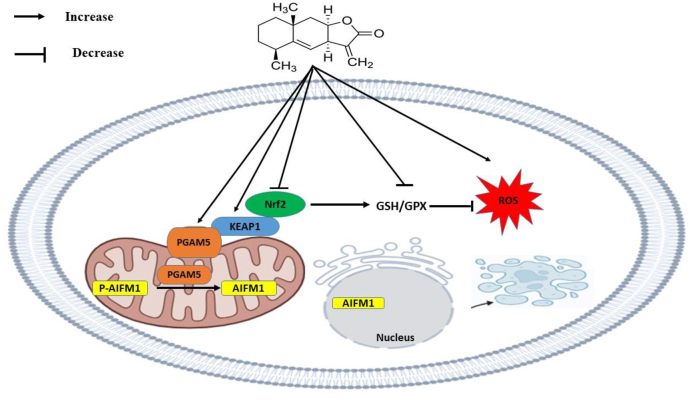
ALT inhibits Nrf2 transcription and elevates ROS production.ALT suppresses the transcription factor Nrf2 expression in SKOV3. Nrf2 stimulates the transcription of numerous cytoprotective genes such as glutathione synthetase and glutathione peroxidase. Downregulation of Nrf2 leads to a decrease in GSH/GPX mRNA levels which enhances ROS. Elevated ROS concentration lead to the dissociation of PGAM5 from KEAP1 and its translocation into the mitochondria. PGAM5 can dephosphorylates AIFM1 at S116. Dephosphorylated AIFM1is transferred to the nucleous and induces oxieptosis cell death pathway.

We investigated ALT effect on KEAP1, Nrf2, PGAM5, AIFM1 and AIFM1 (Ser-116), phospho-specific protein levels by western blot analysis. It was noted that the decrease in the Nrf2 protein content coincided with an increase in the KEAP1 protein content. (Fig. 4 B, D). On the other hand, protein levels of PGAM5was elevated (Fig. 4 F). These findings with Real-time polymerase chain reaction (RT-PCR) test results indicated a relationship between these three components, so the increase in the expression of KEAP1 and PGAM5 is coupled with the decrease in the expression of Nrf2 (see Fig. 5).

We take a look at the effect of ALT on p-AIFM1/AIFM1 ratio by western blot analysis. ALT dephosphorylated pS116-AIM1 in a dose dependent manner and at the same time the AIFM1 concentration was increased (Fig. 4 C, E).

## 5 Discussion

Because of serious side-effects associated with conventional anti-cancer chemotherapy, exploring novel effective anti-cancer treatments that can exactly and effectively eradicate cancer cells is of paramount importance. In the latest years, a variety of herbal materials have been distinguished that induce cell death, particularly in cancer cells through excessive accumulation of ROS [[38], [39], [40], [41]]. According to previous studies ATL promotes ROS definitely in cancer cells by reducing glutathione (GSH) [8,12,42,43]. GSH and GPX are the most important antioxidant systems of the cells that have a crucial role in detoxification of ROS and restoration of several irreversible oxidative damages to main cellular elements such as proteins, nucleic acids and lipids [44]. In this experiment, mRNA expression levels of GSH/GPX were decreased in ALT treated groups except at 16 μM, GSH mRNA expression level was increased. This may be due to the fact that at moderate levels of ROS, expression of antioxidant enzymes increases. Down regulation of GSH/GPX gene expression leads to oxidative stress. Intracellular ROS levels were firmly piloted by various scavenging systems. However, the molecular pathways behind the anti-tumor effects of ATL through ROS overproduction have remained unclear. In this study, we looked into the anti-cancer effect of ATL through oxeiptosis in human SKOV3 ovarian cancer cells. Missense mutations in Nrf2 or mutations of KEAP1, blocks the KEAP1 and Nrf2 interaction and consequently, Nrf2 concentration increases and translocates to the nucleous and activates an antioxidant response system which reduces cancer cells death. The results of the current study are in consistent with earlier research [8,12,17], and it was found that ALT decreased mRNA and protein expression levels of Nrf2 in SKOV3 ovarian cancer cells in a dose dependent manner. (Fig.s. 3 A, Fig. 4B).

Our experiments showed that the reduction in the Nrf2 expression, lead to a decrease in GSH/GPX gene expression and an overproduction of ROS.

Numerous studies have shown that the Keap1 decrease in variety of cancers, including breast, ovarian, liver, non-small cell lung, colorectal, liver, gallbladder, head and neck squamous cell carcinoma [[45], [46], [47], [48], [49]]. The findings of this study indicated that the treatment of SKOV3 cancer cells with ALT increased KEAP1 mRNA and protein levels (Fig. 3, Fig. 4 D), but Nrf2 protein level was decreased (Fig. 4 B). KEAP1 as an endogenous inhibitor of Nrf2 regulates the intracellular concentration and stability of Nrf2. These results lead to the conclusion that ALT increases the amount of ROS in SKOV3 cells via boosting keap1 and decreasing Nrf2 expression.

Under the normal physiological ROS conditions, PGAM5 location is at the external part of outer mitochondrial membrane (OMM) while AIFM1 anchors to the inner mitochondrial membrane (IMM). However, harmful quantities of intracellular ROS, induce KEAP1 and PGAM5 segregation, causing PGAM5 to localize in the mitochondrial lumen where directly targets its substrate AIFM1. Subsequently, PGAM5 dephosphorylates AIFM1 at serine 116. Dephosphorylated AIFM1 translocate into the nucleus and triggers DNA fragmentation [36,50,51].

This study revealed that ALT induce ROS production in SKOV-3 cancer cells, which resulted in oxeiptosis cell death pathway activation. Based on the results of the current real-time and western blot analysis results, increasing the PGAM5 expression causes AIFM1 to be dephosphorylated and activated.

PGAM5 exerts a non-inflammatory function and has central importance within the oxeiptosis pathway which serves as a marker and is a vital regulator of the oxeiptosis pathway. It was showed that SKOV3 ovarian cancer cells treatment with ALT (64 μM) significantly increased mRNA and protein levels of PGAM5 (Fig.s. 3E and Fig. 4 F). The western blot analysis confirmed that PGAM5 up-regulation is associated with a significant increase in dephosphorylated AIFM1 protein rate (Fig. 4 E). The primary challenge of researches is understanding the relationship between ALT induced ROS overproduction and oxeiptosis, which eventually results in the death of SKOV3 ovarian cancer cells.

The western blot analysis showed that up-regulation of PGAM5 and dephosphorylated AIFM1 was associated with down-regulation of phosphorylated AIFM1 (Fig. 4 F, E and C).

The western blot analysis confirmed that PGAM5 up-regulation is associated with a significant decrease in AIFM1 (Ser-116) phosphor specific protein rate (Fig. 4 C). Previous studies have indicated that AIFM1 transfers into the nucleus and mediates chromatin degradation. These data demonstrated that ALT modulates oxeiptosis which encompasses KEAP1, PGAM5 and AIFM1 through ROS accumulation. The main issue of the researchers is to investigate the correlation between ALT induced ROS and oxeiptosis that may institute a defense mechanism to interact and control progression of cancers by focusing on oxidative stress.

This study showed the potential clinical application and novel molecular mechanism of ATL for optimizing the ovarian cancer, SKOV-3 cells therapy. The discovery that the anti-tumor actions of ALT are related to ROS overloads and the oxeiptosis pathway is groundbreaking and will revolutionize the way that tumors are treated.

## 6 Conclusions

In this work, the researchers focused on the anti-tumor effects of ATL, in human SKOV-3 cancer cell line. In addition, the involved molecular mechanisms and the targeted signaling pathways were examined. ATL effectively inhibited the cell development and proliferation of ovarian cancer cells through GSH/GPX depletion and ROS elevation. ROS elevation was followed by PGAM5 up regulation that dephosphorylated AIFM1 at serine 116 residue and induced oxeiptosis cell death pathway. These results suggest that ALT is a promising herbal drug that may be utilized for the treatment of ovarian cancer.

## Declaration of competing interest

The authors report no conflicts of interest. The authors alone are responsible for the content and writing of the paper.

## Data Availability

Data will be made available on request.
